# Validation of a Model for Predicting Magnesium Concentration in Women with Preeclampsia: A Retrospective Cohort Study

**DOI:** 10.1155/2024/1178220

**Published:** 2024-03-12

**Authors:** Erik Holmström Thalme, Magnus Frödin-Bolling

**Affiliations:** ^1^Department of Women's Health, Värnamo Hospital, Region Jönköpings län, Kvinnokliniken, Värnamo sjukhus, SE-331 85 Värnamo, Sweden; ^2^Department of Gynecology & Obstetrics, Karolinska University Hospital, 171 76 Stockholm, Sweden

## Abstract

**Objective:**

To validate a model for predicting magnesium concentration in magnesium sulfate treatment in preeclampsia.

**Design:**

Retrospective cohort study. *Setting*. Three secondary care hospitals, one accepting neonates from gestational week 28 + 0. *Population*. Women with preeclampsia undergoing magnesium sulfate treatment. Subjects initially received Zuspan treatment (4 g bolus and 1 g/h maintenance dose), commonly increased by individual titration. *Main Outcome Measures*. Difference in mean between measured and predicted magnesium concentration. Proportion of women reaching target concentration (>2 mM) in 25 h.

**Results:**

56 women were included, with 356 magnesium measurements available. Mean magnesium concentration was 1.82 mM. The prediction model overestimated magnesium concentration by 0.10 mM (CI 0.04–0.16) but exhibited no bias for weight, creatinine, or treatment duration. Weighted mean infusion rate was 1.22 g/h during 30 hours. Overall success rate in reaching target concentration was 54%, decreasing to 40% in women > 95 kg. Overall success rate at 8 hours was 11%. No toxic concentrations were found.

**Conclusions:**

Zuspan regimen is very safe, but slow to reach therapeutic concentrations—despite efforts of individual titration. Success rate is lower in heavy women, which is of particular importance considering their predisposition to develop preeclampsia. The validated pharmacokinetic model performs well and may be used to individually tailor treatment from the outset.

## 1. Introduction

Preeclampsia is a hypertensive disease affecting 2-8% of all pregnancies with associated edema, placental insufficiency, kidney and liver dysfunction, hemolysis, coagulopathy, and seizures—referred to as eclampsia [1]. Eclampsia is a rare, but potentially fatal complication of preeclampsia. The reported incidence of eclampsia is 1.6 to 10 per 10 000 deliveries in developed countries, whereas it is 50 to 151 per 10 000 deliveries in developing countries [2].

Diagnostic criteria for preeclampsia have changed from elevated blood pressure and proteinuria to a less strict definition of hypertonia and any of several organ dysfunctions, such as anaemia or thrombocytopenia, elevated liver enzymes, central nervous symptoms, proteinuria or elevated creatinine, or foetal growth restriction [3–5].

Magnesium sulfate treatment is described as early as 1933 [6]. In the last decades of the 20^th^ century, magnesium sulfate treatment became less common, due to concerns of magnesium toxicity, and the belief that anticonvulsant drugs were equally efficacious in preventing eclampsia [7].

The mechanism behind neuroprotection in magnesium treatment is not fully understood but is believed to stem from calcium antagonism, blocking overactivation of NMDA receptors and inhibiting inflammatory cytokine response—both factors in a second phase of brain insults [8, 9]. Magnesium sulfate has been tried in other forms of neuroprotection, e.g., stroke and cardiac arrest, with no protective effect found [8, 10].

During the 1950s, Zuspan in Ohio, USA, and Pritchard in Texas, USA, introduced standardised magnesium sulfate treatments. Zuspan advocated a regime of intravenous bolus and maintenance treatment, whilst Pritchard favoured intramuscular bolus and repeat injections [11, 12]. These regimens persist today—Zuspan in high-resource settings and Pritchard in low-resource settings.

The tentative therapeutic range of serum magnesium (2.0–3.0 mM) stems from measurements in successful cases of this era, whilst the threshold of toxicity as measured by loss of patellar reflex (3.5 mM) was established in 1940 [12–14].

In the 2002 Magpie trial (MAGnesium sulfate for Prevention of Eclampsia), designed to evaluate the effects of magnesium sulfate on pregnant women with preeclampsia and their babies, there was a marked reduction in seizures for mothers given magnesium sulfate rather than placebo, regardless of whether treatment is started before or after delivery and irrespective of any previous anticonvulsant therapy. The relative risk of maternal death was 0.55, and 0.42 for eclamptic seizures, favouring the treatment arm [15]. The trial did not measure serum magnesium concentration, and the participating women's body weight and creatinine level were not recorded [15]. Zuspan and Pritchard treatments were used interchangeably.

Since 2002, obesity rates have soared worldwide and are expected to continue to increase [16, 17]. Increased weight increases distribution volume, and thus time to achieve steady state concentration. Obesity is a pronounced risk factor for developing preeclampsia, making it imperative to ascertain that obese women receive adequate magnesium treatment [18, 19].

To clarify the pharmacokinetics of magnesium sulfate in preeclampsia, prospective measurements using a 4 g bolus and 2 g/h maintenance dose were performed in a tertiary centre US cohort [20]. From these measurements, a population pharmacokinetic (PK) model was developed [21].

The body mass index among women giving birth in our health care region is lower than the population used in developing the pharmacokinetic model [21, 22]. Thus, we hypothesised that body weight is lower among women treated with magnesium sulfate in our region and therefore sought to perform an external validation of the PK model. Since preeclampsia is a major cause of preterm delivery and we do not treat extremely preterm neonates, there might also be a difference in patient selection causing gestational age at treatment to start to be higher in our population [23].

The rationale for validating this particular model is that it used a mixed model—decreasing the risk of overfitting model to data, and that the population is well-characterised [20, 21]. A secondary aim of the study was to evaluate the proportion of women in our historical cohort reaching the target serum magnesium of >2 mM.

## 2. Methods

The women studied were treated in the same healthcare region during 2011–2021. Hospitals in the region have two delivery wards without an associated neonatal ward, and one central hospital receiving neonates born in gestational week 28 and later.

Our common treatment guideline magnesium sulfate in preeclampsia is based on Zuspan treatment—4 g bolus dose and maintenance infusion of 1 g/h. In addition, magnesium concentration is measured 1 h after start and changes in maintenance infusion, and if <2 mM maintenance dose is increased by 0.25 g/h. Treatment is recommended to continue 24 h postpartum, but no upper time limit is provided.

Cases to be screened were selected from the electronic medical records by ICD-10 diagnoses: O14.1, O14.2, O14.9, and O15. The sole criterium for inclusion was receiving magnesium treatment. Exclusion criteria were as follows: no recent creatinine measurement; no well-defined start of treatment; no recent body weight measurement; only one measurement of magnesium; and other cause for magnesium treatment. Individuals were allowed to appear several times, each pregnancy recorded as a separate case.

For each case, baseline creatinine and body weight measurements were extracted. Starting time for magnesium bolus, administered bolus dose, starting time for maintenance treatment, and subsequent changes in maintenance dose were extracted from electronic medical records. After validation, time stamps were normalised on a case basis to time since (first) bolus. Each patient had their individual concentration tables calculated using the two-compartment PK model [21].

The PK library used was pmxTools, an R language PK differential equation implementation [24]. The pretreatment concentrations of serum magnesium were unknown and assumed to be 0.74 mM as in the PK cohort [21]. This is in line with other baseline measurements in preeclampsia [25]. Individual concentration tables were predicted using a resolution of 5 minutes, taking changes in infusion rate into account. The measured magnesium concentrations were time-matched to those predicted by the PK model.

### 2.1. Statistical Analysis

Power calculations were made for detection of a difference between estimated and recorded magnesium concentration of 0.2 mM at 1 hour of treatment. The point in time was chosen due to the relatively higher importance of detecting discrepancies in high concentrations to avoid toxicity, such as following the bolus infusion. Using a mean population prediction of 1.43 mM at this time point, the effect size is large, indicating that at least 17 women needed to be included.

All statistical analyses were performed in R [26]. Zuspan treatment produces a concentration peak after bolus infusion, followed by a trough at the start of maintenance infusion, and a second peak when it approaches a steady state at around 24 hours of treatment [21]. Measurements of serum magnesium were divided to reflect this into four groups: early group (<4 h), intermediate (4–16 h), late (17–25), and extended (25–60 h). The extended group was designed to investigate potential toxic concentrations in longer than normal treatment. The subgroups were compared with regard to maintenance dose and measured magnesium concentration.

Overall model performance was assessed by comparing mean predicted and measured concentration. The absolute prediction error for time-matched pairings was compared as a function of weight, creatinine, and treatment duration by using simple linear regression. Absolute prediction error was preferred to relative error due to being more intuitive, and thus clinically valuable. We studied model calibration by plotting measured concentration as a function of predicted concentration.

Eclamptic seizures are very rare. As a proxy for treatment effect, a magnesium target > 2 mM is used. As time is of great importance in severe preeclampsia, we opted to investigate the proportion of women reaching target concentration related to duration of treatment. Thus, Kaplan-Meier event plots were produced for the proportion of women reaching a target concentration of 2.0 mM in the first 25 h of treatment. A proportion of cases reaching target concentration by 8 and 25 hours were recorded, representing an early protective effect and protection in long-lasting cases. Event curves were further stratified by relative body weight and creatinine to explore the effect of patient characteristics on treatment outcome. Differences between groups were assessed using the log-rank test.

To evaluate the prevalence of potentially toxic magnesium concentrations, we reviewed each case with any measurement > 3.5 mM, noting any action taken, repeated sampling, and whether it was likely to be an aberrant concentration or error in measurement.

## 3. Results

**Figure 1:**
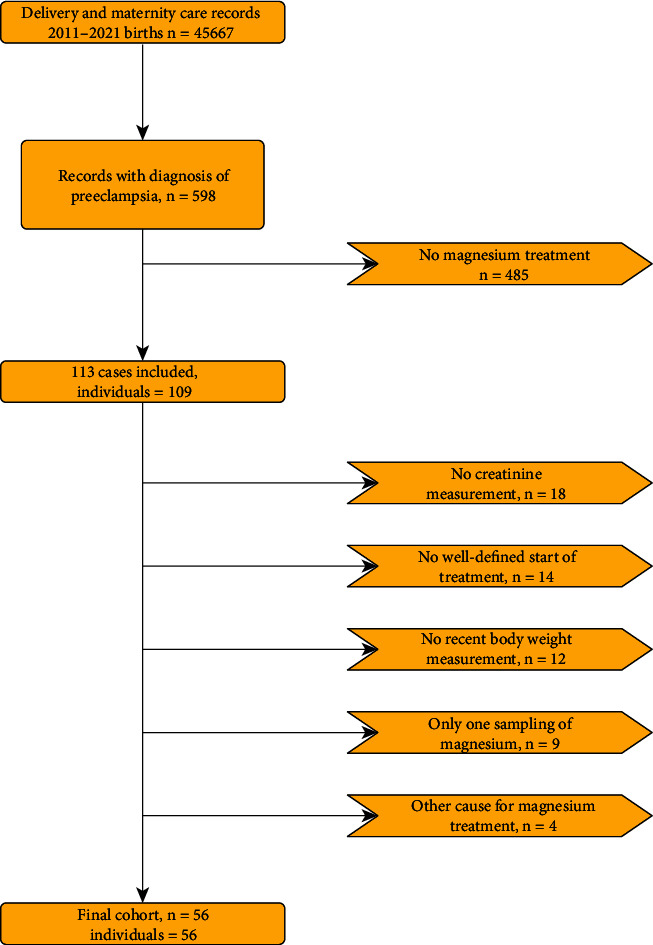
Study flow diagram.

From a total of 45667 delivery records, 598 carried a diagnosis of preeclampsia and were screened for inclusion. Inclusion criteria yielded 113 cases, of which 56 remained after exclusion criteria were applied. Details are provided in Figure 1. Demographics of the final cohort are provided in Table 1.

**Figure 2:**
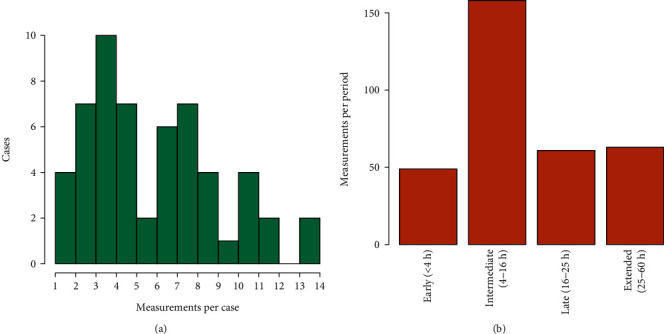
(a) Number of magnesium measurements per case. (b) Distribution of measurements per time period.

In total, 356 measurements of magnesium concentrations had been recorded, at least two and at most fourteen per case; the exact distribution per case is shown in Figure 2(a) and per time period in Figure 2(b). Mean magnesium concentration was 1.82 mM, ranging from 0.73 to 7.28. Six measurements were outliers, all determined to be erroneous measurements due to an unexpectedly high and improbable measurement, subsequently followed by previous or expected levels. Omitting these outliers, the maximum magnesium concentration was 3.06 mM.

Furthermore, we could determine the prevalence of potentially toxic measurements (0.16%), of which none proved to be correct, as proven by renewed blood sampling.

There was a significant increase in infusion speed from the early (1.03 g/h) to intermediate (1.43 g/h) period, but there were no changes in infusion speed thereafter. Magnesium levels continued to increase significantly from early (1.50 mM) to intermediate (1.74 mM) to late (1.99 mM) measurements, but no increase was seen from the late to extended group.

**Figure 3:**
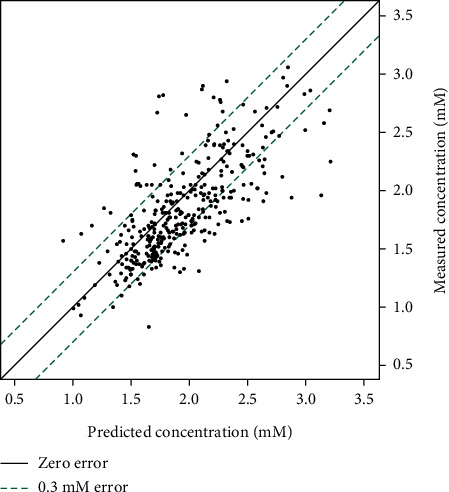
Calibration diagram. With visual aid lines.

Mean measured concentration was 0.10 mM (CI 0.04–0.16) lower than mean time-matched prediction. There were no significant differences in prediction error between the time categories. Furthermore, prediction error did not correlate to weight or creatinine.  Figure 3 shows the calibration curve, exhibiting excellent prediction.

**Figure 4:**
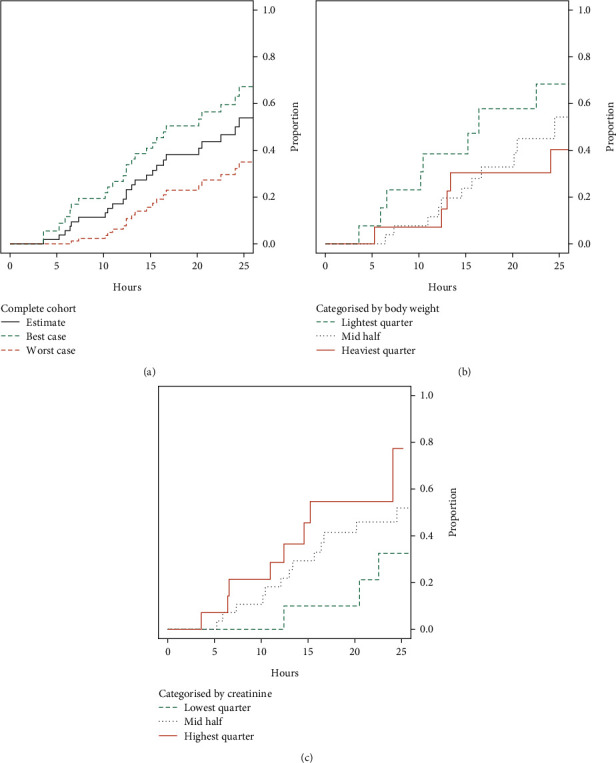
(a) Kaplan-Meier plot for the proportion of women reaching concentration > 2 mmol/L within 25 h, with modelled 95% confidence interval. (b) Success rate plot subgrouped by body weight. (c) Success rate subgrouped by serum creatinine.

The proportion of women reaching >2 mM in 8 and 25 hours is 11% and 54%, respectively. The event curve with confidence intervals is displayed in Figure 4(a). Stratifying by body weight, as shown in Figure 4(b), suggested a difference between groups. At 25 hours, 68% of the lowest body weight quartile had reached target concentration, as opposed to 40% among highest quartile cases. Log-rank test resulted in *p* = 0.08.

Conversely, among the quartile with highest creatinine levels, 78% reached target concentration in 25 hours, as opposed to 33% in the quartile with lowest creatinine measurements. The event curve is visualised in Figure 4(c), log-rank test generating *p* = 0.04.

## 4. Discussion

Our study found a good predictive capability of the pharmacokinetic model. There was a statistically significant difference in prediction vs outcome of +0.10 mM; however, the study was not designed nor powered to evaluate its clinical impact. In a clinical setting, when using a potentially very toxic drug. Overestimation is preferable to underestimation. The model performed well at all concentrations, and without any bias identified.

Compared to the model population, the women in this study had a lower BMI (31.6 vs 34.8; CI -1.18 to -5.22) and creatinine (61.3 vs 72.5; CI -4.51 to -17.88). However, there were no significant differences in body weight (CI -11.05 to 0.86) or gestational week (CI -0.18 to 2.32).

No toxic concentrations were found, demonstrating the safety of the Zuspan regime. Conversely, despite employing additional individual titration, only 54% of cases reached target concentration after standard 24-hour treatment. During the initial stabilising phase in the first 8 hours, only 11% of cases reached target concentration. These findings suggest that either a larger bolus dose or a higher maintenance dose could decrease time to target concentration. As noted by Du et al., a higher maintenance dose risks reaching toxic concentrations, especially if treatment is extended past 24 hours [21].

Additionally, it was found that low creatinine levels severely affected chances of reaching target concentration. As magnesium is eliminated renally, high renal filtration is expected to lead to increased elimination of magnesium, and thus lower serum concentrations. A similar trend was seen for body weight, with higher body weight leading to lower serum concentrations. The study was not fully powered for the stratified analyses, yielding less than 10 events for each stratification quartile. Additionally, nonproportional hazard is a recognised cause for loss of power in log ratio-testing [27]. This study adds to previous ones that have demonstrated a detrimental effect of increasing weight on success rate [20, 28], and increasing success rates in obesity when using increased dosage [29].

The exact range of therapeutic concentration in magnesium sulfate treatment is unknown, and many clinics use Zuspan treatment irrespective of weight. Considering that Zuspan treatment is proven to be efficacious, the therapeutic threshold might be lower than 2.0 mM [21].

One study examined previous trials for a dose-response relationship between magnesium dose and risk for eclampsia, finding that higher dose regimes could further decrease eclampsia risk compared to Zuspan treatment [30]. Using a validated prediction model for magnesium treatment makes high-dose regimens safer by excluding patients at risk for toxic concentrations from treatment, alternately allowing individualised maintenance doses for targeting higher concentrations.

Study limitations are primarily the historical nature of the cohort making this a retrospective study. As all events were recorded in the medical records under standard treatment conditions, i.e., not a prospective study, timing cannot be presumed to be exact, but the possible timing error is small compared to the treatment duration. The strengths of this study lie in its generalisability as it is an unfiltered view of women having received magnesium sulfate treatment. Furthermore, the diversity in infusion rate and frequent rate changes in the cohort provided a realistic test bed for validating the model, and indicating its general safety for use.

## 5. Conclusion

In this historical cohort, magnesium sulfate treatment with using a 4 g bolus and a minimum maintenance dose of 1 g/h produced no toxic concentration and thus did not necessitate additional monitoring with respect to magnesium sulfate treatment. On the contrary, only 54% of treated women reached target concentration > 2.0 mM within 25 hours, falling even lower among women with high body weight or low creatinine. Calculating individual bolus and maintenance doses could be used to improve treatment outcomes and simultaneously decrease blood sampling. Further, the cohort of 56 cases with 356 magnesium measurements validated an external pharmacokinetic model for magnesium sulfate treatment, proving that individualised treatment is feasible—only requiring body weight and serum creatinine level.

## Figures and Tables

**Table 1:** Demographics of final cohort.

	1^st^ quartile	Median	3^rd^ quartile
Age (years)	27	29	32
Gestational age (weeks + days)	33 + 1	35 + 5	38 + 3
Weight (kg)	73	84	95
Creatinine (*μ*mol/L)	51	57	69
Body mass index	26.5	31.2	36.3
Treatment duration (h)	23.7	29.6	46.0
Weighted mean infusion rate during treatment (g/h)	1.00	1.22	1.44

## Data Availability

The clinical and laboratory data used to support the findings of this study are included within the article. The ethical approval restricts access to data to the group level.
